# Early Introduction and Delayed Dissemination of Pandemic Influenza, Gabon

**DOI:** 10.3201/eid1904.111925

**Published:** 2013-04

**Authors:** Sonia Etenna Lekana-Douki, Augustin Mouinga-Ondémé, Dieudonné Nkoghe, Christian Drosten, Jan Felix Drexler, Mirdad Kazanji, Eric M. Leroy

**Affiliations:** Centre International de Recherches Médicales de Franceville, Franceville, Gabon (S.E. Lekana-Douki, A. Mouinga-Ondémé, D. Nkoghe, E.M. Leroy);; Ministère de la Santé Publique, Libreville, Gabon (D. Nkoghe);; Bonn Medical Centre Institute of Virology, Bonn, Germany (C. Drosten, J.F. Drexler);; Institut Pasteur de Bangui, Bangui, Central African Republic (M. Kazanji);; Institut de Recherche pour le Développement, Montpellier, France (E.M. Leroy)

**Keywords:** Gabon, surveillance network, influenza, pH1N1, circulation, pandemic, viruses

## Abstract

Active surveillance in health care centers in Gabon during 2009–2011 detected 72 clinical cases of pandemic (H1N1) 2009 (pH1N1). We found that pH1N1 virus was introduced in mid-2009 but spread throughout the country in 2010. Thus, Gabon was also affected by pH1N1.

In April 2009, a pandemic strain of influenza A (H1N1) (pH1N1) virus emerged in Mexico and the United States; the World Health Organization declared a pandemic alert on June 11, 2009 (*1*,*2*). This virus was responsible for a large outbreak with thousands of cases in the Reunion Islands and in several French tropical Pacific islands during July–October 2009 (*3*). The circulation and public health effects of pH1N1 virus are largely unknown in Africa, with the exception of South Africa and Kenya, which were heavily affected by disease outbreaks during 2009 and 2010 (*4*–*6*). Other pH1N1 cases were reported in several countries of North, West, and East Africa and in Madagascar (*7*,*8*). 

In the humid tropical forest of Central Africa, a study demonstrated the circulation of influenza virus in Cameroon during 2007–2008 (*9*); another reported cases of pH1N1 in Cameroon in 2009 (*10*). A sentinel surveillance program for influenza in Kinshasa, Democratic Republic of the Congo, during 2009–2011 reported several cases of pH1N1 (*11*).

Gabon is a typical humid, tropical, forested country in Central Africa, with 1,517,685 inhabitants and a surface area of 270,000 km^2^. The country has a short dry season during January–February, a long rainy season during March–May, a long dry season during June–September, and a short rainy season during October–December. We report the results of a large surveillance study for pH1N1 in Gabon during a 2-year period, July 2009–June 2011.

## The Study

**Figure 1:**
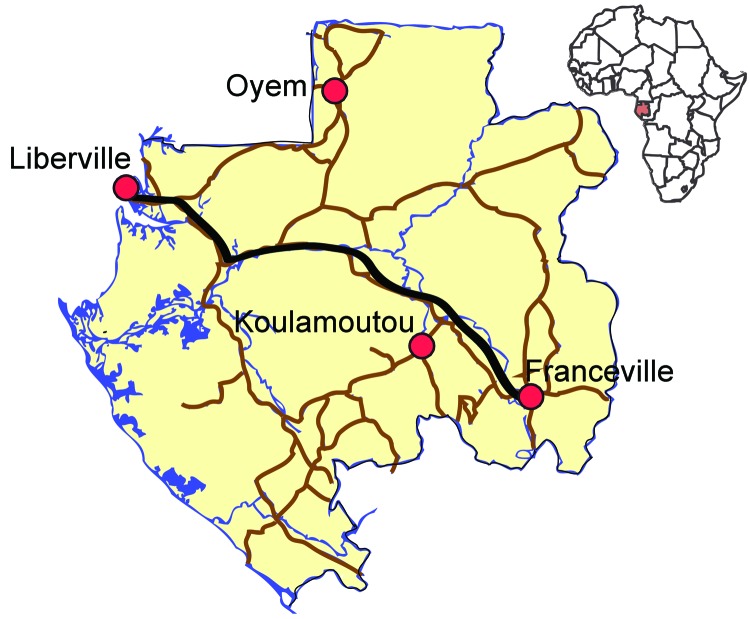
Towns in the influenza sentinel network in Gabon. Libreville was chosen as a typical urban community; Franceville, in the southeast, represents a savannah/forested rural region of 100,000 inhabitants; and Oyem (35,241 inhabitants) and Koulamoutou (16,270 inhabitants), in the north and south, respectively, represent forested rural regions.

Surveillance for influenza-like illness (ILI) was performed during July 2009–June 2011 in the capital city of Gabon, Libreville, and in 3 other towns in rural Gabon (Franceville, Oyem, and Koulamoutou) (Figure 1). ILI was defined as fever (>38°C) and runny nose, cough, or sore throat. Study participants were enrolled at 3 health care centers in Libreville and at the regional hospitals in the other towns; all patients who visited these health centers for ILI were systematically sampled. Individual oral consent was obtained from patients for nasal sampling. 

Epidemiologic data (name, age, sex, and travel history during the month before onset) and clinical data were collected for each patient. Nasal swabs were sent each week to Centre International de Recherches Médicales de Franceville for analysis. Real-time reverse transcription PCR (RT-PCR) was used to detect pH1N1, seasonal influenza A (H1N1 and H3N2), and seasonal influenza B viruses (*12*). Specimens positive for pH1N1 virus were also tested by specific quantitative PCR for the following common respiratory viruses: adenovirus, respiratory syncytial virus, human metapneumovirus, parainfluenzavirus (PIV) 1–4, enterovirus, rhinovirus, parechovirus, and human coronavirus (HCoV; strains OC43, 229E, NL63, and HKU1). Testing protocols are available on request from the authors. Patients who had laboratory-confirmed influenza were contacted several months after diagnosis to determine outcome.

**Figure 2:**
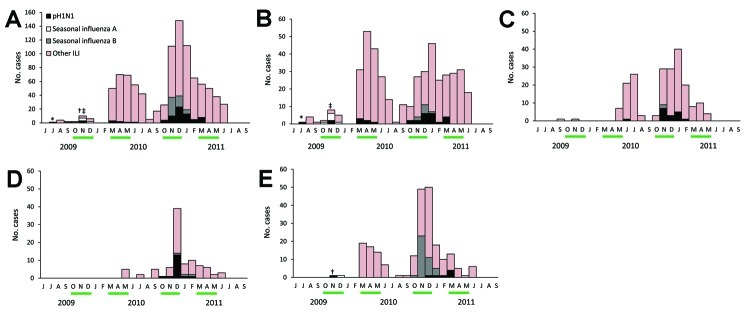
Clinical and laboratory-confirmed cases of pandemic (H1N1) 2009 (pH1N1), seasonal influenza A (H1N1 and H3N2), seasonal influenza B, and other influenza-like illnesses (ILI), Gabon, July 2009–June 2011. Bars below chart indicate rainy seasons. *First imported case; †second imported case; ‡first indigenous cases. A) Gabon; B) Libreville; C) Oyem; D) Koulamoutou; E) Franceville.

Nasal swab specimens were collected from 966 patients with influenza-like symptoms during July 2009–June 2011: 445 from Libreville, 202 from Oyem, 94 from Koulamoutou, and 225 from Franceville (Table). Median patient age was 1.66 years (range 10 days–82 years); 81% of these patients were <4 years of age, and 19% were 4–82 years of age. The M:F sex ratio was 1.02. The number of cases of ILI increased during the 2 rainy seasons and decreased during the 2 dry seasons (Figure 2), which is consistent with a study showing an increase of the number of influenza cases during the rainy seasons in Senegal (*13*).

**Table:** Demographic characteristics of patients and distribution of influenza viruses and other influenza-like illnesses, Gabon, July 2009–June 2011*

Patient and illness data	Influenza virus types				Other†	Total no. patients
	pH1N1	A	B	pH1N1 + A + B		
Sex, no. patients						
M	33	4	23	60	427	487
F	39	4	28	71	408	479
Median age, y (range)	2 (2 mo–49 y)	45 (9–50 y)	2 (3 mo–41 y)	2 (2 mo–50 y)	1.58 (10 d–82 y)	1.66 (10 d–82 y)
Age group, no. patients						
0–23 mo	30	0	20	50	444	494
2–4 y	25	0	17	42	219	261
>4 y	17	5	10	32	147	179
Illness, by year and town						
2009						
Libreville	3 (33)	6 (67)	0	9	12	21
Franceville	1 (50)	1 (50)	0	2	0	2
Koulamoutou	0	0	0	0	0	0
Oyem	0	1 (100)	0	1	1	2
Total	4 (33)	8 (67)	0	12	13	25
2010						
Libreville	16 (70)	0	7 (30)	23	224	247
Franceville	1 (3)	0	34 (97)	35	135	170
Koulamoutou	15 (94)	0	1 (6)	16	42	58
Oyem	11 (85)	0	2 (15)	13	105	118
Total	43 (49)	0	44 (51)	87	506	593
2011						
Libreville	11 (92)	0	1 (8)	12	165	177
Franceville	6 (60)	0	4 (40)	10	43	53
Koulamoutou	2 (50)	0	2 (50)	4	32	36
Oyem	6 (100)	0	0	6	76	82
Total	25 (78)	0	7 (22)	32	316	348
Total						
Libreville	30 (68)	6 (14)	8 (18)	44	401	445
Franceville	8 (17)	1 (2)	38 (81)	47	178	225
Koulamoutou	17 (85)	0	3 (15)	20	74	94
Oyem	17 (85)	1 (5)	2 (10)	20	182	202
Total	72 (55)	8 (6)	51 (39)	131	835	966

*Values are no. (%) cases except as indicated. pH1N1, pandemic (H1N1) 2009; A, seasonal influenza A; B, seasonal influenza B. †Influenza-like illnesses other than pH1N1 or seasonal influenza A or B. Age data were missing for 32 patients in this category.

Among the 966 cases of ILI, 131 (13.6%) were determined to be caused by an influenza virus: 72 (55%) pH1N1, 8 (6%) seasonal influenza A (H1N1 and H3N2), and 51 (39%) influenza B (Table; Figure 2). No deaths caused by pH1N1 were reported during the study period. For the 72 patients infected with pH1N1 virus, median age was 2 years (range 2 months–49 years); 76.4% of these patients were <4 years of age, and 23.6% were 4–49 years of age. The M:F sex ratio was 0.85. 

Only 18 patients with pH1N1 harbored another respiratory virus; this finding suggests that pH1N1 virus infection was responsible for the symptoms in all pH1N1 virus–infected patients. Among patients with pH1N1, we found co-infections with PIV1 (n = 1), PIV3 (n = 2), PIV4 (n = 1), HCoV 229E (n = 1), HCoV OC43 (n = 1), respiratory syncytial virus (n = 7), and adenovirus (n = 5).

The first laboratory-confirmed pH1N1 case (case 1), in a tourist who resided in the Reunion Islands, was diagnosed on July 26, 2009 (Figure 2, panel A). On his trip to Gabon, he had made changeovers in Mauritius and South Africa, 2 countries heavily affected by pH1N1. The patient’s symptoms lasted ≈1 week. The second laboratory-confirmed case (case 2) was detected in Franceville 4 months later, during the short rainy season (Figure 2, panels A, E). ILI developed in this patient 3 days after his arrival in Franceville from France, which was also heavily affected by pH1N1 during this time.

The first 2 autochthonous cases were diagnosed on November 26, 2009 (cases 3), 1 week after the second imported case of pH1N1, during the short rainy season. Subsequently, 29 autochthonous cases were detected in Libreville, 17 in Oyem, 17 in Koulamoutou, and 7 in Franceville (Table). Libreville was the first town with detected pH1N1 cases during the rainy season and also had the highest number of pH1N1 cases (Table). The first autochthonous case was detected in Oyem in early June 2010; during the 2010 short rainy season, several pH1N1 cases were detected in Oyem, indicating pH1N1 virus dissemination throughout Gabon by that time. A total of 85% of the influenza cases in Oyem were pH1N1 (Table). A similar pattern of pH1N1 was observed in Koulamoutou and Franceville during the short rainy season (Figure 2, panels D, E). The percentage of pH1N1 among all influenza cases in Franceville increased from 3% in 2010 to 60% in 2011 (Table). We detected no cases of influenza, including pH1N1, in the provinces of Oyem and Koulamoutou during the first half of 2010. 

Seasonal influenza A was diagnosed in Gabon only during September–December 2009: 6 cases in Libreville, 1 case in Oyem, and 1 case in Franceville. During the short rainy season in 2010, the incidence of influenza B increased: 8 cases in Libreville, 2 cases in Oyem, 3 cases in Koulamoutou, and 38 cases in Franceville (Figure 2; Table). We detected no cases of co-infection with pH1N1 virus and either seasonal influenza A or influenza B viruses.

## Conclusions

Our data suggest that pH1N1 virus was introduced in Gabon just before July 2009, during the first pandemic peak in the Americas and Europe. However, this early introduction did not result in continuous virus circulation in the rest of the country until the short rainy season in 2010. Only during the 2011 season was there a noteworthy increase in case numbers compatible with a pandemic wave, suggesting a notable time lag relative to that for other countries. Our findings indicate that rural tropical countries such as Gabon may serve as reservoirs for later spread of pH1N1 virus within the country and into other countries (*14*,*15*).
